# The consumer quality index (CQ-index) in an accident and emergency department: development and first evaluation

**DOI:** 10.1186/1472-6963-12-284

**Published:** 2012-08-28

**Authors:** Nanne Bos, Leontien M Sturms, Augustinus JP Schrijvers, Henk F van Stel

**Affiliations:** 1 Julius Center for Health Sciences and Primary Care, University Medical Center Utrecht, Utrecht, The Netherlands; 2 Dutch Network for Emergency Care, Tilburg, The Netherlands

**Keywords:** Factor analysis, statistical, Emergency medical services, Patient experiences, Patient satisfaction, statistics and numerical data, Questionnaires, standards, Health care surveys

## Abstract

**Background:**

Assessment of patients’ views are essential to provide a patient-centred health service and to evaluating quality of care. As no standardized and validated system for measuring patients’ experiences in accident and emergency departments existed, we have developed the Consumer Quality index for the accident and emergency department (CQI A&E).

**Methods:**

Qualitative research has been undertaken to determine the content validity of the CQI A&E. In order to assess psychometric characteristics an 84-item questionnaire was sent to 653 patients who had attended a large A&E in the Netherlands. Also, fifty importance questions were added to determine relevance of the questions and for future calculations of improvement scores. Exploratory factor analysis was applied to detect the domains of the questionnaire.

**Results:**

Survey data of 304 (47%) patients were used for the analysis. The first exploratory factor analysis resulted in three domains based on 13 items: ‘Attitude of the healthcare professionals’, ‘Environment and impression of the A&E’ and ‘Respect for and explanation to the patient’. The first two had an acceptable internal consistency. The second analysis, included 24 items grouped into 5 domains: ‘Attitude of the healthcare professionals’, ‘Information and explanation’, ‘Environment of the A&E’,’Leaving the A&E’ and ‘General information and rapidity of care’. All factors were internal consistent. According to the patients, the three most important aspects in healthcare performance in the A&E were: trust in the competence of the healthcare professionals, hygiene in the A&E and patients’ health care expectations. In general, the highest improvement scores concerned patient information.

**Conclusions:**

The Consumer Quality index for the accident and emergency department measures patients’ experiences of A&E healthcare performance. Preliminary psychometric characteristics are sufficient to justify further research into reliability and validity.

## Background

Healthcare services have shown an increasing interest in the quality of care they provide [1]. After clinical outcome evaluations, evaluations based on the patient’s perspective have become more prominent since the introduction of patient-centred care [2]. International organisations such as the Organization for Economic Cooperation and Development (OECD) and the World Health Organization (WHO), have emphasised the importance of the patient’s perspective in the evaluation of healthcare delivery. National and cross-national comparisons of patients’ experiences are important for identifying areas in need of improvement [3]. Patients’ experiences provide information on which healthcare professionals, patients, and health-insurance companies may base their decisions. Furthermore, it enables the government and the Health Care Inspectorate to monitor the quality of healthcare. Finally, the standardized measurement of patients’ experiences enables evaluations of research of research projects.

It is preferable to measure patients’ experiences rather than their satisfaction, as they have shown to be more objective and to yield more detailed information for quality improvement [4]. One theory is that satisfaction is a multi-dimensional concept, partly based on expectations and personal preferences. This complicates the objective measurement of the quality of care. When a product fails to match expectations, the quality will be judged as unsatisfactory [5].

In the Netherlands, the Consumer Quality Index (CQ-index), a standardized method for developing surveys and measuring healthcare quality from the patient’s perspective, was introduced in 2006 in order to promote patient-centred care. In order to obtain reliable and valid questionnaires, the development process has been prescribed in a manual and it is guided and controlled by a scientific advisory board. The content validity is ascertained during a qualitative phase which includes a literature search, interviews with experts, and patient focus groups. After this phase a pilot study on the CQ-index should be performed to determine internal consistency. The CQ-index is characterised by combining patients’ experiences with the relative importance of each experience item resulting in a list of priorities for improvement of quality of care. Several CQ-indices for a variety of community services, care settings and condition-specific patients’ groups have been developed, such as the rheumatoid arthritic questionnaire, the cataract questionnaire, the hip and knee questionnaire, and breast-cancer questionnaire [2,6,7]. Different patient groups turned out to have different priorities, which stressed the need for specific questionnaires [8]. In emergency medicine, two CQ-indices for general practitioners (office hours and out-of-hours), and a CQ-index for maternity services are available. The CQ-indices for ambulance services and dispatch centres are under development. This study completes the set of questionnaires for emergency services with the development of the CQ-index for the accident and emergency department.

In the Netherlands, general practitioners are positioned as gatekeepers, also in the case of emergency care. Emergency care by GPs is provided from local GP-practices during working hours, and out-of-hours in regional GP-cooperatives [9]. Patients need to consult their GP for referral to accident and emergency departments (A&Es) in hospitals. However, the number of self-referrals to A&Es is growing [10]. Patients transported by ambulance in need of emergency care are brought directly to the A&E [11,12]. A&Es are often the place where patients form their first impression of a hospital and a positive experience may influence decisions about future visits and personal patient recommendations [13]. Measuring the quality of care in the A&E as experienced by patients may provide valuable information, for instance for identifying areas in need of improvement.

The goal of this study is to develop and pilot test a CQ-index for the A&E department (CQI A&E). This questionnaire aims to measure healthcare performance in the A&E as experienced by the patient.

## Methods

### Qualitative and constructive phase

The prescribed CQ-index guidelines were applied during the development of the CQI A&E [14,15]. The first phase of the development is a qualitative phase. The aim of this phase is to detect all relevant quality aspects of healthcare performance in the A&E. We carried out a literature search in Pubmed, including a search for existing questionnaires, and interviews with three experts, in order to compose a topic list for focus group discussions with patients about healthcare performance in the A&E. For the focus groups, a consecutive sample of 177 patients treated in the A&E at the University Medical Center Utrecht, aged 18 and older, with known postal address and phone number, were sent an invitation by postal mail to participate, in the first week after their A&E attendance. In a subsequent step, all patients were called and invited a second time to participate in a patient focus group. Seventeen patients confirmed their participation. Two researchers acted as moderators during the focus group discussions. After the focus groups the first draft questionnaire was defined. This draft was sent to ten patients. Within one week, cognitive interviews were performed by telephone in order to ensure that the questions were relevant, unambiguous, understandable and useful to patients, and whether patients had experienced any problems during self-completion of the paper questionnaire. Unclear questions in the CQI A&E were rephrased. Afterwards the CQI A&E consisted of 84 questions divided into 9 categories: General; Before arrival in the A&E; Reception desk A&E; Health professionals in the A&E; Pain; Examination and treatment; Leaving the A&E; General A&E; About you. 52 questions out of the total of 84 questions were constructed as so called ‘experience questions’. The other questions included ‘skip or go to’ items, opinion questions and demographic questions (Additional file 1).

### Importance study

An importance study was undertaken to determine the relative importance of the items in the questionnaire to patients visiting the A&E. Firstly, importance scores were used to decide whether a question should be retained or deleted prior to the factor analysis. Secondly, importance scores are necessary for calculating improvement scores. For each experience question a corresponding importance question was formulated. For example: ‘Was the signposting to the A&E of the hospital a problem?’ with the corresponding importance question ‘How important is the signposting to the A&E of the hospital to you?’. This resulted in a temporary set of 50 extra importance questions in the CQI A&E. Importance questions of two experience questions were unclearly phrased or difficult to understand, and therefore left out of the importance study.

### Psychometric phase

The questionnaire was pilot tested in the psychometric phase to assess the psychometric properties. The three phases are presented in Table 1.

**Table 1:** Three development phases of the CQI A&E

**Qualitative phase**	→ Aim: The detection of quality aspects of healthcare performance in the A&E
	Literature search
	Expert interviews
	Patient focus group discussions
**Constructive phase**	→ Aim: The construction of relevant, unambiguous, understandable and useful questions
	Cognitive interviews with patients
	Importance study
**Psychometric phase**	→ Aim: The assessment of the psychometric properties of CQI A&E
	Pilot test

### Patients

For the pilot test all 653 patients who visited the A&E of a large urban hospital in the course of one week in January 2010, were included. The hospital was centrally located in the Netherlands. 38,000 patients visit the A&E annually. The A&E treats patients in need of urgent care, except for multiple trauma patients, who are referred to specialized trauma centres. Patients who attended to the A&E with a known postal address and no reported death were eligible.

The paper questionnaire and covering letter were sent by postal mail within one week after the visit to the A&E. Up to three reminders were sent to non-respondents: after 1, 4 and 6 weeks. The recipients were able to return the questionnaire in a postage paid envelope. The study protocol was approved by the Medical Ethical Committee of the University Medical Center, Utrecht.

### Data analysis

The hospital registration system provided data on gender, age, referral to A&E (ambulance, general practitioner, self-referred, other), day and time of the visit, triage code, symptoms of which the patients complained (abdominal pain, traumatic injuries, shortness of breath, collapse, chest pain, arrhythmia, malaise/fever, stroke, infection, intoxication, other). The respondents’ gender and age profile was compared to the total sample and to non-respondents using a Chi square or *t*-test, in order to assess whether it was representative. Questionnaires which had been filled in by someone other than the respondent and questionnaires with more than fifty percent of the answers missing, including skip (or ‘go to’) instructions after the questions, were not used for analysis.

### Data quality and exploratory factor analysis

The data set was first analysed in order to identify item response rates and frequency distributions. Questionnaire items were excluded from further analysis if they had an item non-response of >10% of expected responses or extreme skew of >90% of responses in the same category (i.e. a ceiling or floor effect). Spearman’s correlation coefficient was calculated to check for correlations between items (r > 0.70). Where items had a negative wording, their scales were reversed to ensure comparability in the analysis. Exploratory Factor Analysis (EFA) was used to group the experience questions. In the first EFA only the 13 experience items with a 4-point Likert scale were included. EFA was performed with oblique rotation [16]. In EFA several criteria need to be fulfilled. The Kaiser-Meyer-Olkin Measure of Adequacy (KMO) is a measure of sampling adequacy (threshold: KMO >0.60). Bartlett's test of sphericity is used to test the null hypothesis that the variables in the population correlation matrix are uncorrelated (threshold: p < 0.05). The Eigenvalue represents the amount of the total variance explained by the factor (threshold: Eigenvalue > 1, also known as the Kaiser criterion). A variety of analyses were performed, whereby options like ‘fixed number of factors yes or no’ and ‘replace missing values by mean yes or no’ were tested. The domains in the final EFA fulfilled the statistical criteria, explained the highest percentage of variance and had a clear interpretation. In a subsequent step, factor loadings were obtained (threshold: factor load > 0.40). We calculated a measure of internal consistency, Cronbach’s alpha (α), in order to estimate the reliability of the reported factors. Cronbach’s alpha coefficients above 0.70 were considered reliable. In this stage of development alpha coefficients between 0.60-0.70 were provisionally accepted. The α of the total factor should not increase by deleting one of the items. Item-total correlation (ITC) had to be higher than 0.40. When following the CQI guidelines, the majority of experience questions were omitted when constructing domains. Therefore, a second EFA was performed, including all 52 experience questions, with response categories on 2-, 3-, and 4-point Likert scales. This was done to prevent loss of content, thereby ignoring one criterion of the CQI guidelines.

### Experience scores, importance scores and improvement scores

The experience scores and importance scores were calculated as means of response categories (i.e. no/a big problem/never/not important = 1, sometimes/of some importance = 2, a bit of a problem = 2.5, a great deal/important = 3, yes/not a problem/always/extremely important = 4). A domain score was computed as the mean of the experience scores of items contributing to the domain [17]. Quality improvement scores were calculated by multiplying the importance scores with the percentages of the negative response categories ‘never’, ‘sometimes’, ‘big problem’ or ‘no’ on the corresponding experience questions. The improvement scores were an estimate for the potential improvement of quality of care and are useful for internal monitoring, whereas domain scores are more relevant for external monitors. Scores above 0.5 may potentially improve quality of care (range: 0–4). All analyses were performed using the statistical software SPSS 17.0.

## Results

### Qualitative and constructive phase

A review of the literature was conducted, using the PubMed database. A search with Mesh major headings ‘Emergency Service, hospital’ AND ‘Consumer satisfaction’ resulted in 364 hits. All abstracts from 1993 until August 2010 were reviewed for quality dimensions and aspects of care in the A&E. In 53 articles quality aspects were described. The two most frequently used questionnaires were the Consumer Emergency Care Satisfaction Scale (CECSS) and the Emergency Department Patient Satisfaction Survey (EDPSS). Together with the most discussed topics in interviews with experts a topic-list was composed, which was used in patient focus groups discussions. Quality aspects for A&E healthcare delivery were: patient history, accessibility, empathy and attitude of healthcare professionals, autonomy, cooperation, waiting time, competence, triage, treatment, communication, information, pain management, discharge management, re-admittance, privacy, environment, global rating, safety, diagnostic tests, rapidity, refreshments, and accompaniment. The quality aspects were used to formulate the questions and compile the draft questionnaire. After cognitive interviews, the questionnaire was adjusted and questions were added or rephrased where necessary. Substantial adjustments were: the question ‘At what time did you visit the A&E?’ was added to the questionnaire; the question ‘Was the accessibility of the A&E a problem? was rephrased to ‘Was the travelling time to the A&E of the hospital a problem?; ‘Did you have to wait a second time after your first contact with a healthcare professional?’ was rephrased to ‘Was your health problem first briefly assessed by a nurse and did you then have to wait again in the waiting room?’; ‘What score would you give the healthcare professionals?’ was deleted; ‘Did you get the care you expected from the A&E?’ was added. Full detailed information of the qualitative and constructive phase were reported and approved by the advisory board of the CQI A&E [18].

### Importance study

Fifty importance questions were used to calculate the most important aspects in healthcare performance in the A&E according to patients. The five most important aspects were: trust in the competence of healthcare professionals (3.66), hygiene in the A&E (3.65), patients’ healthcare expectations (3.65), patients’ healthcare needs (3.64) and being taken seriously by healthcare professionals (3.63) (Table 2). The five least important items were: information on the order in which patients were treated (2.54), availability of refreshments (2.53), information on an admission letter for the general practitioner (2.50), pleasant atmosphere in the waiting room (2.57) and having to tell the same story about the health problem (2.61). Importance score are ranged from 0–4.

**Table 2:** Importance scores (I) with corresponding quality improvement scores (Q) and corresponding experience scores (E)

**No.**	**Quality aspect**	**I**	**Q**	**E**
1	Trust in competence of healthcare professionals	3.66	0.20	3.70
2	Hygiene in the A&E	3.65	0.30	3.42
3	Patients’ healthcare expectations	3.65	0.23	3.56
4	Patients’ healthcare needs	3.64	0.32	3.58
5	Being taken seriously by healthcare professionals	3.63	0.10	3.81
6	Being taken seriously by the reception staff member at the reception desk	3.57	0.10	3.85
7	Cooperation between healthcare professionals	3.57	0.21	3.59
8	Consistency of the provided information	3.57	0.10	3.84
9	Clarity of explanations of results of examinations	3.55	0.52	3.40
10	Rapidity of the treatment	3.54	0.59	3.33
11	Listening to patients by healthcare professionals	3.53	0.12	3.77
12	Availability of a parking space near the A&E	3.52	**1.05**	2.75
13	Information by the healthcare professionals on danger signals to watch out for after leaving the A&E	3.51	**1.58**	2.66
14	Clarity of explanations of the health problem	3.48	0.36	3.53
15	Feeling safe in the A&E	3.47	0.11	3.73
16	Assessment by the acuity of the patients’ problem	3.46	0.81	3.65
17	Finding the A&E in the hospital	3.46	0.28	3.88
18	Information by healthcare professionals on readmission in case of health problems	3.44	**1.13**	3.02
19	Explanation of the aim of new medication	3.41	0.53	3.40
20	Information on side-effects of the medication	3.39	**2.40**	1.92

*I* importance score (range: 1–4), *Q* quality improvement score (range: 0–4) *E* experience score (range: 1–4).

### Psychometric phase

#### Patients

368 out of 653 patients (56%) returned the questionnaire. Two uncompleted questionnaires were excluded, as were 52 questionnaires which had been filled in by someone other than the patient, for instance by the representatives of patients aged 0–11 years. The dataset for the analysis contained 304 questionnaires (47%). Patients’ characteristics are presented in Table 3. No differences between respondents and non-respondents were found for gender, age, referral, day and time of the visit or symptoms. A significant difference was found for the triage code (p = 0.01). Respondents were triaged in more urgent categories than non-respondents. 34% of the respondents were admitted to a hospital ward after their visit to the A&E.

**Table 3:** Characteristics of the study sample

	**Respondents**		**Non-respondents**	
	Mean (SD)	N	Mean (SD)	N
**Age** (years)	51.4 (21.6)	304	49.3 (24.2)	291
**Gender**	%		%	
Male	52.3	159	49.1	143
Female	47.7	145	50.9	148
**Day and time of attendance**				
Weekday 8:00am – 5:00pm	54.6	166	50.9	148
Weekend day 8:00am – 5:00pm	18.1	55	18.2	53
Out of hours 5:00pm – 12:00am	18.8	57	19.6	57
Out of hours 12:00 am – 8:00pm	8.6	26	11.3	33
**Referral**				
Ambulance	18.4	56	24.1	70
General Practitioner	38.2	116	27.1	79
Self-referred	34.5	105	35.7	104
Other	8.9	27	13.1	38
Triage code *				
Red	0		0	0
Orange	23.2	66	16.4	43
Yellow	39.1	111	37.0	97
Green	37.7	107	45.8	120
Blue	0	0	0.8	2
Missing		20		27
**Symptoms**				
Abdominal pain	10.6	30	9.4	26
Traumatic injuries	35.9	102	40.6	112
Shortness of breath	8.8	25	8.0	22
Collapse	4.9	14	2.5	7
Chest pain	9.9	28	9.1	25
Arrhythmia	5.6	16	1.1	3
Malaise/fever	6.3	18	6.9	19
Stroke	0.7	2	2.2	6
Infection	2.5	7	2.2	6
Intoxication	1.1	2	2.2	6
Other	13.7	39	15.9	44
Missing		20		15
**After A&E**				
Admitted to hospital	34.3	101	N/A	N/A
Discharged to home	62.3	184	N/A	N/A
Other	3.4	10	N/A	N/A
Missing		9		

* p < 0.05 significant difference between respondents and non respondents

*N/A* Not applicable.

#### Data quality

The item ‘access to results of previous visit’ and the item ‘pain control by healthcare professionals’ had a non-response >10%. Extremely skewed items (>90%) were the items ‘signposting to the A&E’, ‘travelling time’, ‘signage in the hospital’ and ‘talking about patients in the presence of the patient’. The five items were left out of the factor analyses. None of the importance items had a remarkably lower score than the average score, or was extremely skewed. Spearman correlations were calculated; none of the correlation coefficients of experience questions or importance questions were above the 0.70 threshold. All experience and importance questions with corresponding frequency distributions on response categories and experience or importance scores are presented in Additional file 2 and Additional file 3.

### First factor analysis and internal consistency

The first EFA, based on 13 items, showed a 3-factor solution with an explained variance of 56%, covering all items (KMO 0.883, Bartlett’s test p < 0.001, N = 298). The first domain measured the quality aspect ‘attitude of healthcare professionals’, the second domain ‘environment and impression of the A&E’ and the third domain ‘respect for and explanation to the patient’ (Table 4). Cronbach’s alpha coefficient of the first domain was 0.85. The alpha coeffiecient of the second domain was 0.60,,and the alpha coefficient of the third domain was 0.42. The item-total correlations of the third factor were below 0.40. The internal consistency did not increase by taking an item out of this third domain.

**Table 4:** Domains, items, and internal consistency of the first factor analysis

**Quality aspect**	**Loading**	**ITC**	**α if item deleted**
**Attitude of healthcare professionals (α = 0.85; n = 278)**			
Politeness of healthcare professionals	0.70	0.58	0.84
Listening to patients by healthcare professionals	0.82	0.74	0.81
The healthcare professionals take time for their patients	0.69	0.64	0.83
Being taken seriously by healthcare professionals	0.88	0.78	0.81
Consistency of the provided information by healthcare professionals	0.57	0.42	0.85
Cooperation between healthcare professionals	0.54	0.52	0.85
Trust in the competence of healthcare professionals	0.82	0.73	0.85
**Environment and impression of the A&E (α = 0.60; n = 289)**			
Hygiene in the A&E	0.82	0.44	0.44
Calm/peaceful A&E	0.69	0.39	0.55
Feeling safe in the A&E	0.69	0.43	0.51
**Respect for and explanation to the patient (α = 0.42; n = 281)**			
Privacy in the treatment room	0.82	0.23	0.38
Involvement in treatment decisions	0.41	0.25	0.42
Clarity of explanations of the health problem to the patient	0.58	0.34	0.20

*ITC* Item-total correlation.

### Second factor analysis and internal consistency

The second analysis was performed on 52 items. Reliability analysis showed that the questionnaire contained four domains with Cronbach’s alpha coefficients above 0.70, and one domain with an alpha coefficient of 0.67. The five domains had an explained variance of 51%. Like the first analysis, this second analysis fulfilled the predefined criteria; Eigenvalue > 1 and KMO = 0.837. However, Bartlett’s test of sphericity was not significant (p = 1.00; N = 298). Domains, items and Cronbach’s alpha coefficients are presented in Table 5. All item-total correlations were above 0.40, with the exception of the item ‘consistency of provided information’. The Cronbach’s alpha of the domain remained the same if the item was left out. The five domains covered 24 items.

**Table 5:** Domains, items, and internal consistency of the second factor analysis

**Quality aspect**	**Loading**	**ITC**	**α if item deleted**
**Attitude of healthcare professionals (α =0.88; n = 165)**			
Patients’ healthcare needs	0.53	0.62	0.86
Politeness of healthcare professionals	0.71	0.67	0.87
Listening to patients by healthcare professionals	0.78	0.77	0.85
Healthcare professionals take time for their patients	0.71	0.65	0.86
Being taken seriously by healthcare professionals	0.87	0.82	0.85
Consistency of the provided information by healthcare professionals	0.52	0.37	0.88
Cooperation between healthcare professionals	0.48	0.53	0.88
Trust in the competence of healthcare professionals	0.77	0.75	0.85
Feeling safe in the A&E	0.57	0.56	0.87
**Information and explanation (α = 0.83; n = 41)**			
Information on treatment	0.67	0.67	0.78
Clarity of explanations of results of examinations	0.49	0.67	0.79
Clarity of explanations (general)	0.65	0.71	0.77
Explanation about how to make an appointment in the policlinic	0.61	0.54	0.82
Information towards attendants	0.56	0.59	0.81
**Environment of the A&E (α = 0.67; n = 159)**			
Pleasant atmosphere in waiting room	0.69	0.51	N/A
Refreshments	0.72	0.51	N/A
**Leaving the A&E (α = 0.67; n = 38)**			
Explanation about new medication	0.66	0.41	0.77
Information on side-effects of the medication	0.71	0.66	0.63
Information on resumption of daily activities	0.51	0.50	0.72
Information on danger signals to watch out for after leaving the A&E	0.67	0.64	0.64
**General information and rapidity of care (α = 0.71; n = 53)**			
Information on the rapidity of the treatment based on acuity of the health problem	0.57	0.52	0.63
Information on the order of treatment	0.69	0.50	0.64
Pain control	0.60	0.50	0.64
Rapidity of the treatment	0.41	0.46	0.67

*ITC* Item-total correlation.

*N/A* Not applicable due to one remaining item after deletion.

#### Quality improvement

For every item the experience score and quality improvement score were computed. Within the top 20 of most important quality aspects, four aspects stood out as far as their improvement potential was concerned: ‘information on side-effects of the medication’, ‘information by healthcare professionals on danger signals to watch out for after leaving the A&E’, ‘information by healthcare professionals on readmission in case of health problems’, and ‘availability of parking space near the A&E’ (Table 2). Three out of four items were dealing with information needs at the end of the A&E visit, and belonged to the 14 items with the highest improvement scores, i.e. a quality improvement score >1; range 0–4 (Table 6).

**Table 6:** Items with quality improvement scores >1 (Q) with corresponding importance scores (I) and corresponding experience scores (E)

**No.**	**Quality aspect**	**I**	**Q**	**E**
1	Information on side-effects of the medication	3.39	2.40	1.92
2	Information on the rapidity of the provided care	3.28	2.20	2.01
3	Information by healthcare professionals on the admission letter for the GP	2.50	2.11	1.47
4	The GP is informed by healthcare professionals	2.85	2.10	1.80
5	Information on the order of treatment	2.54	2.07	1.53
6	Involvement in treatment decisions	3.12	1.76	2.38
7	Healthcare professionals help to control the pain	3.12	1.75	2.27
8	Information by healthcare professionals on danger signals to watch out for after leaving the A&E	3.51	1.58	2.66
9	Information by the reception staff member on procedures in the A&E	3.07	1.51	2.50
10	Information by healthcare professional on resumption of daily activities	3.30	1.35	2.65
11	Pleasant atmosphere in waiting room	2.55	1.31	2.49
12	Information by healthcare professional on readmission in case of health problems	3.44	1.13	3.02
13	Healthcare professionals ask to consent to treatment	3.08	1.10	2.76
14	Availability of a parking space near the A&E	3.52	1.05	2.75

*I* importance score (range: 1–4), *Q* quality improvement score (range: 0–4), *E* experience score (range: 1–4).

## Discussion

The aim of this study has been to construct and test a questionnaire to measure patients’ experiences with the accident and emergency department, according to the guidelines of the Consumer Quality index [15]. This first version of the Consumer Quality index for the accident and emergency department (CQI A&E) consisted of 84 questions. 52 questions were phrased as ‘experience questions’. The response rate (47%) of the pilot test was comparable to other postal surveys involving A&E patients [14,19]. However, disease-specific CQ-indices showed better response rates (68%–84%) [2,6,7,20,21]. To determine the construct of the questionnaire, two exploratory factor analyses were performed. The first analysis was performed including thirteen items. Three domains were constituted (‘attitude of healthcare professionals’, ‘environment and impression of the A&E’, ‘respect for and explanation to the patient’). A second analysis comprising all 52 experience questions was performed with the aim of including more questions on experiences deemed important by patients. Five domains ‘attitude of healthcare professionals’, ‘information and explanation’, ‘environment of the A&E’, ‘leaving the A&E’, and ‘general information and rapidity of care’ were constructed, covering 24 items. Two out of three domains in the first EFA were internally consistent, whereas all domains in the second EFA were internally consistent. Internal consistency of domains increases by increasing the number of respondents. Despite of lower response numbers in the domains of the second EFA, the internal consistency exceeded the internal consistency of the first EFA. The percentage of explained variance of the five domains decreased five percent compared to the explained variance of the three domains of the first analysis.

Two main goals for CQI-data can be distinguished. The first goal is to compare quality of care between healthcare providers. A strict (following the CQI-guidelines), statistically correct EFA was performed in order to generate the information needed for making a valid comparison between A&Es. Researchers, the Health Care Inspectorate, health-insurance companies, hospital boards and the Ministry of Health are the intended users of these outcomes. However, the main customer in healthcare is the patient. Therefore, the second goal is to acquire the information needed for quality improvement within a healthcare institution. To this end, an alternative EFA was performed. To include more content of the questionnaire in the domains, more questions were added. We think this information may help A&E managers and others to start evaluating quality improvement projects. Both goals represent a different way of constructing domains in questionnaires. Following all criteria in the CQI guidelines, domains are constructed using the perspective of a reflective measurement model [22,23]. Only items with a 4-point Likert scale were included. Items that did not fit into any domain, and domains that did not fulfil the statistical criteria, were omitted [24]. The qualitative phase was carried out in order to detect all aspects related to healthcare performance in the A&E. Each aspect is a unique part of the provided care and together they form the construct ‘quality of care’. To end up with a few statistically related items neglects the broad range of the aspects. Therefore, a formative measurement model may be better suited to construct domains in experience questionnaires. The latter theory concerns the construction of domains based on content and not solely on strict statistical criteria. In the second analysis, we did not try to construct domains solely from a formative perspective, but we tried to include as many experience questions as possible, while still achieving internally consistent and interpretable domains. We included all domains that came up in the second EFA, thereby doubling the content of the questionnaire included in internally consistent domains. Although we only relaxed one of the criteria of CQI-guidelines, we think that these domains are better suited for quality improvement and that they are also suited for benchmarking. The improvement scores provide concrete tailor-made information, which can be helpful for management and staff.

In accordance with most CQ-indices, the domains on communication, information, attitude of healthcare providers (often within the communication domain) and the environment of the health service, are part of the CQI A&E. Domains regarding accessibility and leaving the organisation are also often found [6,7,20,25]. There are a lot of similarities to other CQI instruments, whereas the similarities with satisfaction questionnaires are few. The A&E satisfaction questionnaire Quality Patient Perspective discussed patient participation [19]. The Swedish A&E Patient Satisfaction Survey revealed 3 factors: caring, teaching and clinical competence [26].

The most important aspects in healthcare performance in the A&E from the patient’s perspective dealt with competence of professionals, hygiene and expectations. It has to be determined whether these importance scores are valid across study populations.

In an English importance study, with 16 participants who had visited the accident and emergency department the most important aspect was confidence and trust in the doctors and nurses. Secondly, ‘being treated with respect and dignity’, and thirdly ‘explanation about condition and treatment’ were important items. Interestingly, waiting time did not feature in the top 20 of most important aspects in our study. As regards the top three of least important aspects, our study concorded with the English study on aspects such as refreshments in the waiting room and not being asked details about the patient’s condition or illness too often. However, in the English study the number of respondents was limited, and inclusion criteria broader.

The study has same limitations. Firstly, we used the pilot study dataset of respondents of only one hospital. In the next phase of the development, the stability of the domain structure will be assessed in a dataset of 20 A&Es, and therefore the presented domains are preliminary outcomes. The discriminative capacity of the CQI A&E will be assessed in that phase as well. Secondly, in this study a consecutive sample was used for the psychometric analysis. All patients who had visited the A&E within an average week were included. The gender and age profile of the respondents was representative for the A&E population of the research hospital. Respondents and non-respondents were comparable as regards age, gender, day or time of attendance and symptoms. Therefore, it is unlikely that the low response rate has affected the outcomes. A significant difference between both groups was found for the triage code. The least severely injured patients (blue triage code) were underrepresented within the respondents group. These patients are often discharged without experiencing all aspects of healthcare performance and perhaps did not think of themselves as a ‘true’ A&E patient. This might have introduced selection bias among respondents.

The study protocol had advantages such as sending the questionnaires to the patient’s home, instead of administering them in person in the A&E, which prevents selection bias caused by healthcare professionals. Also, all patients received the questionnaire within one week after their A&E visit, which limits the recall bias. However, patients’ symptoms might have evoked recall bias.

## Conclusions

The Consumer Quality index for the accident and emergency department measures patients’ experiences of A&E healthcare performance. Preliminary psychometric characteristics of the CQI A&E are good, but further research on reliability and validity is needed. Depending on the viewpoint, exploratory factor analysis results in two or five internally consistent domains. The 5-domain structure seems preferable, as this includes more content of the questionnaire while maintaining internal consistency. Furthermore, the improvement scores of each item provide information that makes it possible to identify aspects that require consideration in order to increase quality of care. The preliminary outcomes and the discriminative capacity have to be confirmed in future research by means of the CQI A&E.

## Abbreviations

CQ-index, Consumer quality index; ED, Emergency Department; CQI ED, Consumer Quality Index for the Emergency Department; EFA, Exploratory factor analysis; KMO, Kaiser-Meyer-Olkin measure of adequacy; ITC, Item total correlation.

## Competing interests

As indicated in the paper, none of the authors have conflicts of interest to disclose.

## Authors’ contributions

NB has participated in the study concept, study design, acquisition of the data, data analysis, data interpretation, drafting the manuscript and critical revision of the manuscript. LS has participated in the study concept, study design, acquisition of the data, data analysis, data interpretation, drafting the manuscript and critical revision of the manuscript. AS has participated in the study concept and study design, critical revised the manuscript, and supervised the study. HS has participated in the study concept, study design, acquisition of the data, data analysis, data interpretation, drafting the manuscript and critical revision of the manuscript. All authors have read and approved the final manuscript.

## Pre-publication history

The pre-publication history for this paper can be accessed here:

http://www.biomedcentral.com/1472-6963/12/284/prepub

## Supplementary Material

Additional file 1 CQI A&E. Click here for file

Additional file 2 Frequency distributions, mean experience scores, and 95% confidence interval of experience questions. Click here for file

Additional file 3 Frequency distributions, mean importance scores, and 95% confidence interval of importance questions. Click here for file
