# Supplementary material for: The consumer quality index (CQ-index) in an accident and emergency department: development and first evaluation
Source: BMC Health Serv Res. 2012 Aug 28;12:284. doi: 10.1186/1472-6963-12-284 (PMC3447703; doi:10.1186/1472-6963-12-284)
Supplement: Additional file 1 — CQI A&E. [file 1472-6963-12-284-S1.doc]

**Appendix A – Frequency distributions, mean experience scores, and 95% confidence interval of experience questions**

| Appendix A – Frequency distributions of experience questions and experience scores (mean and 95% confidence interval) | | | | | | |
| --- | --- | --- | --- | --- | --- | --- |
|  | |  | **Experience score** | | | |
|  | |  |  | **95% CI** | | |
|  | | **N** | **Mean** | **Lower** | | **Upper** |
| **Attitude of healthcare professionals** | |  |  |  | |  |
| Q36 | Did you receive the help you needed? |  |  |  | |  |
|  | No, not at all=2.2%; a bit=6.6%; a great deal=22.1%; yes, completely=69.1% | 181 | 3.58 | 3.48 | | 3.69 |
|  |  |  |  |  | |  |
| Q42 | Were the healthcare professionals polite? |  |  |  | |  |
|  | No, not at all=0%; a bit=0.7%; a great deal=9.4%; yes, completely=89.9% | 298 | 3.90 | 3.87 | | 3.94 |
|  |  |  |  |  | |  |
| Q43 | Did the healthcare professionals listen to you attentively? |  |  |  | |  |
|  | No, not at all=0.3%; a bit=3.0%; a great deal=16.2%; yes, completely=80.5% | 297 | 3.77 | 3.71 | | 3.83 |
|  |  |  |  |  | |  |
| Q44 | Did the healthcare professionals have enough time for you? |  |  |  | |  |
|  | No, not at all=1.0%; a bit=3.7%; a great deal=28.9%; yes, completely=66.4% | 298 | 3.61 | 3.54 | | 3.68 |
|  |  |  |  |  | |  |
| Q45 | Did the healthcare professionals take you seriously? |  |  |  | |  |
|  | No, not at all=0.7 %; a bit=2.0%; a great deal=12.8%; yes, completely=84.6% | 298 | 3.81 | 3.76 | | 3.87 |
|  |  |  |  |  | |  |
| Q48 | Did the healthcare professionals give you contradictory information? |  |  |  | |  |
|  | No, not at all=88.7%; a bit=8.6%; a great deal=1.0%; yes, completely=1.7% | 291 | 3.84 | 3.78 | | 3.90 |
|  |  |  |  |  | |  |
| Q49 | Did the healthcare professionals cooperate with each other? |  |  |  | |  |
|  | No, not at all=3.2%; a bit=2.8%; a great deal=25.8%; yes, completely=68.2% | 283 | 3.59 | 3.51 | | 3.67 |
|  |  |  |  |  | |  |
| Q50 | Do you trust the expertise of the healthcare professionals in the A&E? |  |  |  | |  |
|  | No, not at all=2.0%; a bit=3.4%; a great deal=17.6%; yes, completely=77.0% | 296 | 3.70 | 3.62 | | 3.77 |
|  |  |  |  |  | |  |
| Q68 | Did you feel safe during your stay in the A&E? |  |  |  | |  |
|  | No, not at all=0%; a bit=3.1%; a great deal=20.7%; yes, completely=76.3% | 295 | 3.73 | 3.67 | | 3.79 |
|  |  |  |  |  | |  |
| Q71 | Would you recommend the A&E to your friends and family? |  |  |  | |  |
|  | Definitely not=2.4%; probably not=5.9%; probably=46.6%; Definitely=45.2% | 290 | 3.26 | 3.16 | | 3.36 |
|  |  |  |  |  | |  |
| Q73 | Did you receive the care you expected from the A&E? |  |  |  | |  |
|  | No, not at all=1.7%; a bit=4.5%; a great deal=30.3%; yes, completely=63.4% | 290 | 3.56 | 3.48 | | 3.63 |
|  |  |  |  |  | |  |
| **Information and explanation** | |  |  |  | |  |
| Q37 | Did the care provider in the treatment room give you information on the steps in your treatment? |  |  |  | |  |
|  | No, not at all=4.0%; a bit=9.0%; a great deal=20.6%; yes, completely=66.4% | 277 | 3.49 | 3.40 | | 3.59 |
|  |  |  |  |  | |  |
| Q39 | Did the care provider explain the results of these tests in an understandable manner? |  |  |  | |  |
|  | No, not at all=7.5%; a bit=7.1%; a great deal=23.0%; yes, completely=62.3% | 239 | 3.40 | 3.29 | | 3.52 |
|  |  |  |  |  | |  |
| Q47 | Did the healthcare professionals explain your health problem in an understandable manner? |  |  |  | |  |
|  | No, not at all=4.2%; a bit=6.2%; a great deal=22.5%; yes, completely=67.1% | 289 | 3.53 | 3.43 | | 3.62 |
|  |  |  |  |  | |  |
| Q63 | Did the healthcare professionals explain how to make a follow-up appointment? |  |  |  | |  |
|  | No, not at all=7.8%; a bit=6.5%; a great deal=6.5%; yes, completely=79.2% | 77 | 3.57 | 3.36 | | 3.78 |
|  |  |  |  |  | |  |
|  |  |  |  |  | |  |
|  |  |  |  |  | |  |
| Appendix A – continued | |  |  |  | |  |
|  |  |  | **Experience score** | | | |
|  |  |  |  | **95% CI** | | |
|  |  | **N** | **Mean** | **Lower** | | **Upper** |
| **Environment of the A&E** | |  |  |  | |  |
| Q69 | If you were accompanied by others (partner/family/friends), did they get information on you? |  |  |  | |  |
|  | No, not at all=9.5%; a bit=7.4%; a great deal=13.6%; yes, completely=69.4% | 242 | 3.43 | 3.31 | | 3.55 |
| Q64 | Was the atmosphere (planning) of the waiting room pleasant? (magazines/television/chairs)? |  |  |  | |  |
|  | No, not at all=20.8%; a bit=30.7%; a great deal=26.6%; yes, completely=21.9% | 192 | 2.49 | 2.34 | | 2.64 |
|  |  |  |  |  | |  |
| Q65 | If you so wished, could you obtain something to eat or drink in the A&E? |  |  |  | |  |
|  | No, not at all=15.8%; a bit=21.1%; a great deal=18.9%; yes, completely=44.3% | 228 | 2.92 | 2.77 | | 3.66 |
|  |  |  |  |  | |  |
| Q66 | Did you find the A&E hygienic? |  |  |  | |  |
|  | No, not at all=2.4%; a bit=5.9%; a great deal=38.8%; yes, completely=52.9% | 289 | 3.42 | 3.34 | | 3.50 |
|  |  |  |  |  | |  |
| **Leaving the A&E** | |  |  |  | |  |
| Q54 | Did the healthcare professionals explain the aim of the new medicines in an understandable manner? |  |  |  | |  |
|  | No, not at all=9.2%; a bit=6.2%; a great deal=20.0%; yes, completely=64.6% | 65 | 3.40 | 3.16 | | 3.64 |
|  | |  |  |  | |  |
| Q55 | Did the healthcare professionals inform you of side-effects to which you had to pay attention? |  |  |  | |  |
|  | No, not at all=56.9%; a bit=13.8%; a great deal=9.2%; yes, completely=20.0% | 65 | 1.92 | 1.62 | | 2.22 |
|  |  |  |  |  | |  |
| Q56 | Did the healthcare professionals tell you when you could resume your usual activities, such as eating or walking? |  |  |  | |  |
|  | No, not at all=28.1%; a bit=12.9%; a great deal=24.5%; yes, completely=34.5% | 139 | 2.65 | 2.45 | | 2.86 |
|  |  |  |  |  | |  |
| Q57 | Did the healthcare professionals tell you which danger signals you should watch out for after leaving the A&E? |  |  |  | |  |
|  | No, not at all=24.9%; a bit=20.1%; a great deal=18.9%; yes, completely=36.1% | 169 | 2.66 | 2.48 | | 2.85 |
|  | |  |  |  | |  |
| **General information** | |  |  |  | |  |
| Q22 | Did the nurse tell you how quickly you needed to be helped with your health problem? |  |  |  | |  |
|  | No, not at all=48.8%; a bit=18.2%; a great deal=16.5%; yes, completely=16.5% | 121 | 2.01 | 1.80 | | 2.22 |
|  |  |  |  |  | |  |
| Q23 | Did the nurse tell you the order you and the other patients in the waiting room would be helped? |  |  |  | |  |
|  | No, not at all=77.2%; a bit=4.4%; a great deal=7.0%; yes, completely=11.4% | 114 | 1.53 | 1.33 | | 1.72 |
|  |  |  |  |  | |  |
| Q33 | Did the healthcare professionals help you to control your pain? |  |  |  | |  |
|  | No, not at all=42.6%; a bit=13.5%; a great deal=18.4%; yes, completely=25.4% | 244 | 2.27 | 2.11 | | 2.42 |
|  |  |  |  |  | |  |
| Q35 | If you needed help, did you receive it as quickly as you wanted? |  |  |  | |  |
|  | No, not at all=5.5; a bit=11.0%; a great deal=28.2%; yes, completely=55.2% | 181 | 3.33 | 3.20 | | 3.46 |
|  |  |  |  |  | |  |
| **Individual items** | |  |  |  | |  |
| Q7 | Was the record (data) of your previous visit to the A&E available? |  |  |  | |  |
|  | No=14.1%; Yes=85.9% | 64 | 3.58 | 3.32 | | 3.84 |
|  |  |  |  |  | |  |
| Q11 | Was the signposting to the A&E of the hospital a problem? |  |  |  | |  |
|  | A big problem=0.9%; a bit of a problem=8.5%; not a problem=90.6% | 213 | 3.85 | 3.78 | | 3.91 |
|  |  |  |  |  | |  |
| Appendix A – continued | |  |  |  | |  |
|  | |  | **Experience score** | | | |
|  | |  |  | **95% CI** | | |
|  | | N | **Mean** | **Lower** | | **Upper** |
| Q12 | Was the travelling time to the A&E of the hospital a problem? |  |  |  | |  |
|  | A big problem=0.0%; a bit of a problem=6.5%; not a problem=93.5% | 217 | 3.90 | 3.85 | | 3.95 |
| Q13 | Was there a problem in finding a parking space near to the A&E? |  |  |  | |  |
|  | A big problem=29.7%; a bit of a problem=23.7%; not a problem=46.6%; | 219 | 2.75 | 2.58 | | 2.93 |
|  | |  |  |  | |  |
| Q14 | Was there a problem in finding the A&E in the hospital? |  |  |  | |  |
|  | A big problem=0.9%; a bit of a problem=6.5%; not a problem=92.6% | 217 | 3.88 | 3.81 | | 3.94 |
|  | |  |  |  | |  |
| Q16 | Did you have enough privacy at the reception counter when you explained your health problem? |  |  |  | |  |
|  | No, not at all=9.7%; a bit=13.3%; a great deal=21.2%; yes, completely=55.8% | 165 | 3.23 | 3.07 | | 3.39 |
|  | |  |  |  | |  |
| Q17 | Was the reception staff member polite? |  |  |  | |  |
|  | No, not at all=0.6%; a bit=1.8%; a great deal=8.5%; yes, completely=89.1% | 165 | 3.86 | 3.79 | | 3.93 |
|  | |  |  |  | |  |
| Q18 | Did the reception staff member treat you seriously? |  |  |  | |  |
|  | No, not at all=0%; a bit=2.4%; a great deal=9.7%; yes, completely=87.9% | 165 | 3.85 | 3.79 | | 3.92 |
|  | |  |  |  | |  |
| Q19 | Did the reception staff give you information on what to expect during your visit to the A&E? |  |  |  | |  |
|  | No, not at all=28.2%; a bit=20.9%; a great deal=23.9%; yes, completely=27.0% | 163 | 2.50 | 2.32 | | 2.68 |
|  | |  |  |  | |  |
| Q25 | Was the total waiting time before you started treatment in the treatment room a problem? |  |  |  | |  |
|  | A big problem=8.2%; a bit of a problem=26.5%; not a problem=65.3%; | 219 | 3.36 | 3.23 | | 3.48 |
|  | |  |  |  | |  |
| Q27 | Was it a problem that you had to wait longer because more serious patients were treated first? |  |  |  | |  |
|  | A big problem=0.0%; a bit of a problem=23.3%; not a problem=76.7% | 30 | 3.65 | 3.41 | | 3.89 |
|  | |  |  |  | |  |
| Q29 | Were you in any pain while you were in the A&E? |  |  |  | |  |
|  | No=16.6%; Yes=83.4% | 290 | 3.50 | 3.37 | | 3.63 |
|  | |  |  |  | |  |
| Q31 | Did you receive medication to reduce the pain? |  |  |  | |  |
|  | No=59.9%; Yes=29.1%; N/A=11.1% | 289 | 2.09 | 1.94 | | 2.25 |
|  | |  |  |  | |  |
| Q34 | Did you have enough privacy during your examination in the treatment room? |  |  |  | |  |
|  | No, not at all=2.0%; a bit=7.7%; a great deal=15.2%; yes, completely=75.1% | 297 | 3.63 | 3.55 | | 3.71 |
|  | |  |  |  | |  |
| Q40 | Could you decide about your treatment? |  |  |  | |  |
|  | No, not at all=36.7%; a bit=19.7%; a great deal=12.8%; yes, completely=30.8% | 289 | 2.38 | 2.23 | | 2.52 |
|  | |  |  |  | |  |
| Q41 | Were you asked to consent to your treatment? |  |  |  | |  |
|  | No=35.7%; Yes=50.9% | 291 | 2.76 | 2.58 | | 2.95 |
|  | |  |  |  | |  |
| Q46 | Did healthcare professionals talk in front of you as if you weren’t there? |  |  |  | |  |
|  | No, not at all=2.7%; a bit=0.7%; a great deal=6.5%; yes, completely=90.1% | 293 | 3.84 | 3.78 | | 3.90 |
|  | |  |  |  | |  |
| Q58 | Did your healthcare professionals tell you who to contact if you were worried about your health problem after leaving the A&E? |  |  |  | |  |
|  | No=32.8%; Yes=67.2% | 174 | 3.02 | 2.81 | | 3.23 |
|  | |  |  |  | |  |
|  |  |  |  |  | |  |
|  |  |  |  |  | |  |
|  | |  |  |  | |  |
|  |  |  |  |  | |  |
| Appendix A – continued | |  |  |  | |  |
|  |  |  | **Experience score** | | | |
|  |  |  |  | **95% CI** | | |
|  |  | **N** | **Mean** | **Lower** | **Upper** | |
| Q60 | Did the healthcare professionals tell you that your general practitioner would be informed about your visit to the A&E? |  |  |  | |  |
|  | No=73.7%; Yes=26.7% | 191 | 1.80 | 1.61 | | 1.99 |
| Q67 | Was the environment in the A&E quiet? |  |  |  | |  |
|  | No, not at all=3.1%; a bit=8.8%; a great deal=28.9%; yes, completely=59.2% | 294 | 3.44 | 3.35 | | 3.53 |
| Q72 | What score would you give the A&E? |  |  |  | |  |
|  | 0=0.3%; 1=0%; 2=0.3%; 3=0.7%; 4=1.7%; 5=2.4%; 6=7.4%; 7=22.0%; 8=42.2%; 9=12.5%; 10=10.5% | 296 |  |  | |  |
