# Supplementary material for: The consumer quality index (CQ-index) in an accident and emergency department: development and first evaluation
Source: BMC Health Serv Res. 2012 Aug 28;12:284. doi: 10.1186/1472-6963-12-284 (PMC3447703; doi:10.1186/1472-6963-12-284)
Supplement: Additional file 2 — Frequency distributions, mean experience scores, and 95% confidence interval of experience questions. [file 1472-6963-12-284-S2.doc]

**Appendix B – Frequency distributions, mean importance scores, and 95% confidence interval of importance questions**

| Appendix B – Frequency distributions of importance questions and importance scores (mean and 95% confidence interval) | | | | | | | | |
| --- | --- | --- | --- | --- | --- | --- | --- | --- |
|  | |  | **Importance score** | | | | | |
|  | |  |  | | **95% CI** | | | |
|  | | **N** | **Mean** | | **Lower** | | | **Upper** |
| 1 | How important is the availability if the record (data) of your previous visit to the A&E to you? |  |  | |  | | |  |
|  | A=6.7%; B=16.7%; C=43.3%; D=33.3% | 282 | 3.03 | | 2.93 | | | 3.13 |
|  | |  |  | |  | | |  |
| 2 | How important is the signposting to the A&E of the hospital to you? |  |  | |  | | |  |
|  | A=3.2%; B=5.3%; C=45.2%; D=46.3% | 283 | 3.35 | | 3.26 | | | 3.43 |
|  | |  |  | |  | | |  |
| 3 | How important is the travelling time to the A&E of the hospital to you? |  |  | |  | | |  |
|  | A=5.7%; B=8.9%; C=54.3%; D=31.2% | 282 | 3.11 | | 3.02 | | | 3.20 |
|  | |  |  | |  | | |  |
| 4 | How important is finding a parking space near to the A&E to you? |  |  | |  | | |  |
|  | A=1.1%; B=4.2%; C=36.3%; D=58.5% | 284 | 3.52 | | 3.45 | | | 3.59 |
|  | |  |  | |  | | |  |
| 5 | How important is finding the A&E in the hospital to you? |  |  | |  | | |  |
|  | A=6.7%; B=16.7%; C=43.3%; D=33.3% | 284 | 3.44 | | 3.37 | | | 3.52 |
|  | |  |  | |  | | |  |
| 6 | How important is having enough privacy at the reception counter when you explain your health problem to you? |  |  | |  | | |  |
|  | A=4.9%; B=14.3%; C=46.2%; D=34.6% | 286 | 3.10 | | 3.01 | | | 3.20 |
|  | |  |  | |  | | |  |
| 7 | How important is the politeness of the reception staff member to you? |  |  | |  | | |  |
|  | A=1.0%; B=7.7%; C=50.0%; D=41.3% | 286 | 3.31 | | 3.24 | | | 3.39 |
|  | |  |  | |  | | |  |
| 8 | How important is it to you that the reception staff member treats you seriously? |  |  | |  | | |  |
|  | A=0.3%; B=1.4%; C=40.6%; D=57.7% | 286 | 3.56 | | 3.49 | | | 3.62 |
|  | |  |  | |  | | |  |
| 9 | How important is it to you that the reception staff gives you information on what to expect during your visit to the A&E? |  |  | |  | | |  |
|  | A=6.0%; B=12.0%; C=52.5%; D=29.6% | 284 | 3.06 | | 2.96 | | | 3.15 |
|  | |  |  | |  | | |  |
| 10 | How important is the waiting time until you first speak to a healthcare professional to you? |  |  | |  | | |  |
|  | A=1.8%; B=15.8%; C=45.1%; D=37.3% | 284 | 3.18 | | 3.09 | | | 3.27 |
|  | |  |  | |  | | |  |
| 11 | How important is it to you that the nurse tells you how quickly you needed to be helped with your health problem? |  |  | |  | | |  |
|  | A=0.7%; B=8.0%; C=54.2%; D=37.1% | 286 | 3.28 | | 3.20 | | | 3.35 |
|  | |  |  | |  | | |  |
| 12 | How important is to you that the nurse tells you the order you and the other patients in the waiting room would be helped? |  |  | |  | | |  |
|  | A=20.8%; B=22.3%; C=40.3%; D=16.6% | 283 | 2.53 | | 2.41 | | | 2.64 |
|  | |  |  | |  | | |  |
| 13 | How important is the second waiting time before treatment to you? |  |  | |  | | |  |
|  | A=5.6%; B=23.6%; C=47.9%; D=22.9% | 284 | 2.88 | | 2.78 | | | 2.98 |
|  | |  |  | |  | | |  |
| 14 | How important is it to you that the total waiting time before treatment is not a problem? |  |  | |  | | |  |
|  | A=8.9%; B=23.3%; C=47.4%; D=20.4% | 270 | 2.79 | | 2.69 | | | 2.90 |
|  | |  |  | |  | | |  |
| 15 | How important is it to you that more serious patients were treated first? |  |  | |  | | |  |
|  | A=0.0%; B=7.0%; C=40.9%; D=52.1% | 286 | 3.45 | | 3.38 | | | 3.52 |
|  |  |  |  | |  | | |  |
| 16 | How important is it to you that healthcare professionals ask you if you are in pain? |  |  | |  | | |  |
|  | A=0.4%; B=5.6%; C=55.1%; D=38.9% | 285 | 3.33 | | 3.26 | | | 3.40 |
| Appendix B – continued | |  |  | | | | | |
|  | |  | **Importance score** | | | | | |
|  | |  |  | | | **95% CI** | | |
|  | | **N** | **Mean** | **Lower** | | | **Upper** | |
| 17 | How important is it to you that you receive medication to reduce the pain? |  |  |  | | |  | |
|  | A=3.2%; B=17.4%; C=48.9%; D=30.5% | 282 | 3.07 | 2.98 | | | 3.16 | |
|  | |  |  |  | | |  | |
| 18 | How important is the help of healthcare professionals to control your pain? |  |  |  | | |  | |
|  | A=1.4%; B=15.5%; C=53.0%; D=30.0% | 283 | 3.12 | 3.03 | | | 3.20 | |
|  | |  |  |  | | |  | |
| 19 | How important is the privacy during your examination in the treatment room to you? |  |  |  | | |  | |
|  | A=1.1%; B=10.9%; C=46.0%; D=42.1% | 285 | 3.29 | 3.21 | | | 3.37 | |
|  | |  |  |  | | |  | |
| 20 | How important is it to you that if you need help, you receive it as quickly as you want? |  |  |  | | |  | |
|  | A=0.7%; B=6.3%; C=33.8%; D=59.2% | 284 | 3.51 | 3.44 | | | 3.59 | |
|  | |  |  |  | | |  | |
| 21 | How important is it to you that you receive the help you need? |  |  |  | | |  | |
|  | A=0.3%; B=1.4%; C=35.5%; D=62.7% | 287 | 3.61 | 3.54 | | | 3.67 | |
|  | |  |  |  | | |  | |
| 22 | How important is it to you that the care provider in the treatment room gives you information on the steps in your treatment? |  |  |  | | |  | |
|  | A=0.0%; B=5.2%; C=54.7%; D=40.1% | 287 | 3.35 | 3.28 | | | 3.42 | |
|  | |  |  |  | | |  | |
| 23 | How important is it to you that the care provider explains the results of these tests in an understandable manner? |  |  |  | | |  | |
|  | A=0.7%; B=3.5%; C=38.0%; D=57.8% | 287 | 3.53 | 3.46 | | | 3.60 | |
|  | |  |  |  | | |  | |
| 24 | How important is it to you that you can decide about your treatment? |  |  |  | | |  | |
|  | A=3.6%; B=15.4%; C=48.2%; D=32.9% | 280 | 3.10 | 3.01 | | | 3.20 | |
|  | |  |  |  | | |  | |
| 25 | How important is it to you that you are asked to consent to your treatment? |  |  |  | | |  | |
|  | A=5.2%; B=12.9%; C=50.7%; D=31.1% | 286 | 3.08 | 2.98 | | | 3.17 | |
|  | |  |  |  | | |  | |
| 26 | How important is the politeness of the healthcare professionals to you? |  |  |  | | |  | |
|  | A=0.3%; B=8.3%; C=51.7%; D=39.6% | 288 | 3.31 | 3.23 | | | 3.38 | |
|  | |  |  |  | | |  | |
| 27 | How important is it to you that the healthcare professionals listen to you attentively? |  |  |  | | |  | |
|  | A=0.0%; B=4.2%; C=40.6%; D=55.2% | 288 | 3.51 | 3.44 | | | 3.58 | |
|  | |  |  |  | | |  | |
| 28 | How important is it to you that the healthcare professionals have enough time for you? |  |  |  | | |  | |
|  | A=0.3%; B=7.3%; C=53.0%; D=39.4% | 287 | 3.31 | 3.24 | | | 3.39 | |
|  | |  |  |  | | |  | |
| 29 | How important is it to you that the healthcare professionals take you seriously? |  |  |  | | |  | |
|  | A=0.0%; B=1.4%; C=35.9%; D=62.7% | 284 | 3.61 | 3.55 | | | 3.67 | |
|  | |  |  |  | | |  | |
| 30 | How important is it to you that the healthcare professionals talk in front of you as if you aren’t there? |  |  |  | | |  | |
|  | A=5.3%; B=7.4%; C=35.0%; D=52.3% | 283 | 3.34 | 3.25 | | | 3.44 | |
|  | |  |  |  | | |  | |
| 31 | How important is it to you that the healthcare professionals explain your health problem in an understandable manner? |  |  |  | | |  | |
|  | A=0.3%; B=3.8%; C=45.8%; D=50.0% | 288 | 3.45 | 3.39 | | | 3.52 | |
|  | |  |  |  | | |  | |
| 32 | How important is it to you that you don’t receive contradictory information of the healthcare professionals? |  |  |  | | |  | |
|  | A=0.4%; B=3.9%; C=33.7%; D=62.1% | 285 | 3.58 | 3.51 | | | 3.64 | |
|  |  |  |  |  | | |  | |
| 33 | How important is the cooperation of the healthcare professionals with each other to you? |  |  |  | | |  | |
|  | A=0.0%; B=2.4%; C=39.0%; D=58.5% | 287 | 3.56 | 3.50 | | | 3.62 | |
| Appendix B – continued | |  |  |  | | |  | |
|  | |  | **Importance score** | | | | | |
|  | |  |  | **95% CI** | | | | |
|  | | **N** | **Mean** | **Upper** | | | **Lower** | |
| 33 | How important is the cooperation of the healthcare professionals with each other to you? |  |  |  | | |  | |
|  | A=0.0%; B=2.4%; C=39.0%; D=58.5% | 287 | 3.56 | 3.50 | | | 3.62 | |
|  | |  |  |  | | |  | |
| 34 | How important is trust in the expertise of the healthcare professionals in the A&E to you? |  |  |  | | |  | |
|  | A=0.0%; B=0.7%; C=35.0%; D=64.3% | 286 | 3.64 | 3.58 | | | 3.69 | |
|  | |  |  |  | | |  | |
| 35 | How important is it to you that you have to tell the same story several times? |  |  |  | | |  | |
|  | A=18.1%; B=21.0%; C=43.8%; D=17.1% | 281 | 2.60 | 2.48 | | | 2.71 | |
|  | |  |  |  | | |  | |
| 36 | How important is it to you that the healthcare professionals explain the aim of the new medicines in an understandable manner? |  |  |  | | |  | |
|  | A=0.0%; B=3.9%; C=52.5%; D=43.7% | 284 | 3.40 | 3.33 | | | 3.46 | |
|  | |  |  |  | | |  | |
| 37 | How important is it to you that the healthcare professionals inform you of side-effects to which you had to pay attention? |  |  |  | | |  | |
|  | A=1.1%; B=8.1%; C=42.1%; D=48.8% | 285 | 3.39 | 3.31 | | | 3.47 | |
|  | |  |  |  | | |  | |
| 38 | How important is it to you that the healthcare professionals tell you when you can resume your usual activities, such as eating or walking? |  |  |  | | |  | |
|  | A=1.8%; B=8.5%; C=51.1%; D=38.7% | 282 | 3.27 | 3.19 | | | 3.35 | |
|  | |  |  |  | | |  | |
| 39 | How important is it to you that the healthcare professionals tell you which danger signals you should watch out for after leaving the A&E? |  |  |  | | |  | |
|  | A=0.4%; B=1.8%; C=45.8%; D=52.1% | 284 | 3.50 | 3.43 | | | 3.56 | |
|  | |  |  |  | | |  | |
| 40 | How important is it to you that your healthcare professionals tell you who to contact if you are worried about your health problem after leaving the A&E? |  |  |  | | |  | |
|  | A=1.8%; B=3.9%; C=45.4%; D=48.9% | 284 | 3.42 | 3.34 | | | 3.49 | |
|  | |  |  |  | | |  | |
| 41 | How important is it to you that the healthcare professionals tell you that your general practitioner will be informed about your visit to the A&E? |  |  |  | | |  | |
|  | A=9.8%; B=19.6%; C=46.0%; D=24.6% | 285 | 2.85 | 2.75 | | | 2.96 | |
|  | |  |  |  | | |  | |
| 42 | How important is it to you that you receive a referral letter for your general practitioner? |  |  |  | | |  | |
|  | A=22.2%; B=24.4%; C=36.6%; D=16.8% | 279 | 2.48 | 2.36 | | | 2.60 | |
|  | |  |  |  | | |  | |
| 43 | How important is it that the healthcare professionals explain how to make a follow-up appointment? |  |  |  | | |  | |
|  | A=8.1%; B=20.1%; C=54.2%; D=17.6% | 284 | 2.81 | 2.72 | | | 2.91 | |
|  | |  |  |  | | |  | |
| 44 | How important is it to you that the atmosphere (planning) of the waiting room is pleasant? (magazines/television/chairs)? |  |  |  | | |  | |
|  | A=14.4%; B=29.8%; C=40.0%; D=15.8% | 285 | 2.57 | 2.46 | | | 2.68 | |
|  | |  |  |  | | |  | |
| 45 | How important is it that if you so wish, you can obtain something to eat or drink in the A&E? |  |  |  | | |  | |
|  | A=12.9%; B=34.3%; C=37.8%; D=15.0% | 286 | 2.55 | 2.44 | | | 2.65 | |
|  | |  |  |  | | |  | |
| 46 | How important is the hygiene in the A&E to you? |  |  |  | | |  | |
|  | A=0.3%; B=3.5%; C=28.2%; D=67.9% | 287 | 3.64 | 3.57 | | | 3.70 | |
|  | |  |  |  | | |  | |
| 47 | How important is an quiet environment in the A&E to you? |  |  |  | | |  | |
|  | A=1.7%; B=17.5%; C=49.0%; D=31.8% | 286 | 3.12 | 3.02 | | | 3.20 | |
| Appendix B – continued | |  |  |  | | |  | |
|  | |  | **Importance score** | | | | | |
|  | |  |  | **95% CI** | | | | |
|  | | **N** | **Mean** | **Upper** | | | **Lower** | |
| 48 | How important is feeling safe during your stay in the A&E to you? |  |  |  | | |  | |
|  | A=0.3%; B=7.0%; C=38.0%; D=54.7% | 287 | 3.47 | 3.40 | | | 3.54 | |
|  | |  |  |  | | |  | |
| 49 | How important is it to you that if you are accompanied by others (partner/family/friends), they get information on you? |  |  |  | | |  | |
|  | A=1.1%; B=13.7%; C=45.6%; D=39.6% | 285 | 3.24 | 3.15 | | | 3.32 | |
|  | |  |  |  | | |  | |
| 50 | How important is it to you that you receive the care you expect from the A&E? |  |  |  | | |  | |
|  | A=0.0%; B=3.5%; C=29.1%; D=67.4% | 285 | 3.64 | 3.57 | | | 3.70 | |
| A=Not important; B=Of some importance; C=Important; D=Extremely important | |  |  |  | | |  | |
