# Supplementary material for: The consumer quality index (CQ-index) in an accident and emergency department: development and first evaluation
Source: BMC Health Serv Res. 2012 Aug 28;12:284. doi: 10.1186/1472-6963-12-284 (PMC3447703; doi:10.1186/1472-6963-12-284)
Supplement: Additional file 3 — Frequency distributions, mean importance scores, and 95% confidence interval of importance questions. [file 1472-6963-12-284-S3.doc]

**Appendix C – CQI A&E**

1. On what date do you complete this questionnaire?

Date:……..-……….-……….

2. On which date did you visit the A&E? (This concerns your most recent visit to the A&E) Date:……..-……….-……….

3. At what time did you visit the A&E?

❒ Between 8:00a.m.-17:00p.m. ❒ Between 5:00p.m.-12:00a.m. ❒ Between 12:00a.m.-8:00a.m.

4. How serious, according to you, was the health problem for which you visited the A&E?

❒ Not serious ❒ Somewhat serious ❒ Serious ❒ Very serious

5. According to you, how quickly should you have been seen?

❒ Not quickly (had no haste) ❒ Urgent (aid necessary within some hours) ❒ Emergency (aid necessary within half an hour) ❒ Life- threatening (each second counts)

6. Have you visited the A&E for the same health problem before?

❒ No 🡪 Go to Question 8 ❒ Yes

7. Was the record (data) of your previous visit to the A&E available?

❒ No ❒ Yes

8. Who referred you to the A&E?

❒ My General Practitioner 🡪 Go to Question 10 ❒ The General Practitioner Cooperatives 🡪 Go to Question 10 ❒ I was brought by an ambulance 🡪 Go to Question 28 ❒ A specialist told me I had to go to the A&E 🡪 Go to Question 10 ❒ Someone else (e.g. a friend, family member, colleague) decided I had to go to the A&E 🡪 Go to Question 10 ❒ I decided myself that I had to go to the A&E

9. What was the most important reason for you to go directly to the A&E?

❒ The seriousness of my health problem ❒ My General Practitioner was not available/telephonically contactable ❒ The General Practitioner Cooperative was not contactable by telephone ❒ I did not know where else to go ❒ Other reason, namely……

10. How did you travel to the A&E?

❒ By car ❒ By taxi, walked or on a bike ❒ By public transport 🡪 Go to question 12 ❒ By ambulance 🡪 Go to question 28

11. Was the signposting to the A&E of the hospital a problem?

❒ A big problem ❒ A small problem ❒ No problem

12. Was the travelling time to the A&E of the hospital a problem?

❒ A big problem ❒ A small problem ❒ No problem

13. Was there a problem in finding a parking place near to the A&E?

❒ A big problem ❒ A small problem ❒ No problem ❒ N/A (came by public transport, taxi, walking or on a bike)

14. Was there a problem in finding the A&E in the hospital?

❒ A big problem ❒ A small problem ❒ No problem

15. Did an A&E reception staff member register you into the A&E?

❒ No 🡪 G*o to question 20* ❒ Yes

16. Did you have enough privacy at the reception counter when you explained your health problem?

❒ No, not at all ❒ A bit ❒ A great deal ❒ Yes, completely

17. Was the reception staff member polite to you?

❒ No, not at all ❒ A bit ❒ A great deal ❒ Yes, completely

18. Did the reception staff member treat you seriously?

❒ No, not at all ❒ A bit ❒ A great deal ❒ Yes, completely

19 Did the reception staff give you information on what to expect during your visit to the A&E?

❒ No, not at all ❒ A bit ❒ A great deal ❒ Yes, completely

20. How long did you have to wait before you first spoke to a care provider?

❒ 0 – 10 minutes ❒ 11 – 30 minutes ❒ 31 – 60 minutes ❒ 1 – 2 hours ❒ 2 – 4 hours ❒ I don’t know (anymore)

21. Was your health problem first briefly assessed by a nurse and then you had to wait again in the waiting room?

❒ No 🡪 G*o to question 25* ❒ Yes

22. Did the nurse tell you how quickly you needed to be helped with your health problem?

❒ No, not at all ❒ A bit ❒ A great deal ❒ Yes, completely

23. Did the nurse tell you the order you and the other patients in the waiting room would be helped?

❒ No, not at all ❒ A bit ❒ A great deal ❒ Yes, completely

24. How long did you have to wait this second time in the waiting room before your treatment started?

❒ I was helped directly ❒5 – 10 minutes ❒ 11 – 30 minutes ❒ 31 – 60 minutes ❒ 1 – 2 hours

❒ 2 – 4 hours ❒ Longer than 4 hours ❒ I don’t know (anymore)

25. Was the total waiting time before you started treatment in the treatment room a problem?

❒ A big problem ❒ A small problem ❒ Not a problem ❒ N/A (I did not wait in the waiting room) 🡪 G*o to question 28*

26. Did you have to wait longer because more serious patients were treated first?

❒ No 🡪 G*o to question 28* ❒ Yes

27. Was it a problem that you had to wait longer because more serious patients were treated first?

❒ A big problem ❒ A small problem ❒ Not a problem

28. Can you indicate on a scale of 0 to 10 how much pain you had on entry to the A&E? 0 means no pain. 10 means the most terrible pain conceivable.

❒ 0 ❒ 1 ❒ 2 ❒ 3 ❒ 4 ❒ 5 ❒ 6 ❒ 7 ❒ 8 ❒ 9 ❒ 10

29. Did the healthcare professionals ask you if you were in pain?

❒ No ❒ Yes

30. Did you tell the healthcare professionals that you were in pain?

❒ No ❒ Yes

31. Did the healthcare professionals in the A&E give you medication to reduce the pain?

❒ No 🡪 G*o to question 33* ❒ Yes ❒ N/A (I wasn’t in pain) 🡪 G*o to question 33*

32. How long after you spoke to the healthcare professionals about your pain did you receive medication to reduce your pain?

❒ Directly ❒1 – 5 minutes ❒ 6 – 10 minutes ❒ 11 – 15 minutes ❒ 16 – 30 minutes ❒ longer than 30 minutes

33. Did the healthcare professionals in the A&E help you to control your pain?

❒ No, not at all ❒ A bit ❒ A great deal ❒ Yes, completely

34. Did you have enough privacy during your examination in the treatment room?

❒ No, not at all ❒ A bit ❒ A great deal ❒ Yes, completely

35. If you needed help, did you receive it as quickly as you wanted?

❒ No, not at all ❒ A bit ❒ A great deal ❒Yes, completely ❒ N/A (I did not need help) 🡪 G*o to question 37*

36. Did you receive the help you needed?

❒ No, not at all ❒ A bit ❒ A great deal ❒ Yes, completely

37. Did the healthcare professionals in the treatment room give you information on the steps in your treatment?

❒ No, not at all ❒ A bit ❒ A great deal ❒Yes, completely ❒ N/A (there were no further steps in my treatment plan)

38. During your visit to the A&E were tests performed such as X-rays, ECG, echo or blood tests?

❒ No 🡪 G*o to question 40* ❒ Yes

39. Did the care provider explain the results of these tests in an understandable manner?

❒ No, not at all ❒ A bit ❒ A great deal ❒ Yes, completely

40. Could you decide about your treatment?

❒ No, not at all ❒ A bit ❒ A great deal ❒ Yes, completely

41. Were you asked to consent to your treatment?

❒ No ❒ Yes ❒ N/A (I was not treated)

42. Were the healthcare professionals polite to you?

❒ No, not at all ❒ A bit ❒ A great deal ❒ Yes, completely

43. Did the care providers listen attentively to you?

❒ No, not at all ❒ A bit ❒ A great deal ❒ Yes, completely

44. Did the care providers have enough time for you?

❒ No, not at all ❒ A bit ❒ A great deal ❒ Yes, completely

45. Did the care providers take you seriously?

❒ No, not at all ❒ A bit ❒ A great deal ❒ Yes, completely

46. Did the healthcare professionals talk in front of you as if you weren’t there?

❒ No, not at all ❒ A bit ❒ A great deal ❒ Yes, completely

47. Did the care providers explain your health problem in an understandable manner?

❒ No, not at all ❒ A bit ❒ A great deal ❒ Yes, completely

48. Did the care providers give you contradictory information?

❒ No, not at all ❒ A bit ❒ A great deal ❒ Yes, completely

49. Did the care providers cooperate with each other?

❒ No, not at all ❒ A bit ❒ A great deal ❒ Yes, completely

50. Do you trust the expertise of the care providers in the A&E?

❒ No, not at all ❒ A bit ❒ A great deal ❒ Yes, completely

51. How often in the A&E did you have to tell your story of your health problem?

❒ 0 times ❒ 1 – 3 times ❒ More than 3 times

52. What happened at the end of your visit to the A&E?

❒ I was taken (admitted) to (*Insert name of institution*) 🡪 Go *to question 64* ❒ I was transferred to another hospital or nursing home 🡪 G*o to question 64* ❒ I went home ❒ I went to a friend or family ❒ Other, namely………..

53. Did the care provider in the A&E prescribe new medication for you?

❒ No 🡪 G*o to question 56* ❒ Yes

54. Did the care provider explain the aim of the new medicines in an understandable manner?

❒ No, not at all ❒ A bit ❒ A great deal ❒ Yes, completely

55. Did the care provider inform you of side-effects to which you had to pay attention?

❒ No, not at all ❒ A bit ❒ A great deal ❒ Yes, completely

56. Did the care provider tell you when you could resume your usual activities, such as eating or walking?

❒ No, not at all ❒ A bit ❒ A great deal ❒ Yes, completely ❒ N/A (I was not restricted in my usual activities)

57. Did you care provider tell you what danger signals to watch out for after your departure from the A&E?

❒ No, not at all ❒ A bit ❒ A great deal ❒ Yes, completely ❒ N/A (my health problem was solved)

58. Did your care provider tell you who to contact if you were worried about your health problem after leaving the A&E?

❒ No 🡪 G*o to question 60* ❒ Yes ❒ N/A (my health problem was solved) 🡪 G*o to question 60*

59. With whom were you to get in touch? (several answers possible)

❒ Own general practitioner ❒ A&E ❒ Another department in (*insert Name Institution*)

❒ Other, namely………..

60. Did the care provider tell you that your general practitioner would be informed about your visit to the A&E?

❒ No 🡪 Go to question 62 ❒ Yes

61. Did you receive a referral letter for your general practitioner?

❒ No ❒ Yes

62. Did you have to make a follow-up appointment in the policlinic of the hospital?

❒ No 🡪 Go *to question 64* ❒ Yes

63. Did the care provider explain how to make this appointment?

❒ No, not at all ❒ A bit ❒ A great deal ❒ Yes, completely ❒ N/A (my appointment was made the A&E care provider)

64. Was the atmosphere (planning) of the waiting room pleasant? (magazines/television/chairs)?

❒ No, not at all ❒ A bit ❒ A great deal ❒ Yes, completely ❒N/A (I did not wait in the waiting room)

65. If you wished it could you obtain something to eat or drink in the A&E?

❒ No, not at all ❒ A bit ❒ A great deal ❒ Yes, completely ❒ I don’t know (anymore)

66. Did you find the A&E hygienic?

❒ No, not at all ❒ A bit ❒ A great deal ❒ Yes, completely

67. Was the environment in the A&E quite?

❒ No, not at all ❒ A bit ❒ A great deal ❒ Yes, completely

68. Did you feel safe during your stay in the A&E?

❒ No, not at all ❒ A bit ❒ A great deal ❒ Yes, completely

69. If you were accompanied by others (partner/family/friends) did they get information about you?

❒ No, not at all ❒ A bit ❒ A great deal ❒ Yes, completely ❒ N/A (I was not accompanied)

70. How long in total was your visit to the A&E?

❒ Shorter than 1 hour ❒ 1 – 2 hours ❒ 2 – 4 hours ❒ 4 – 8 hours ❒ 8 – 12 hours ❒ Longer than 12 hours

71. Would you recommend the A&E to your friends and family?

❒ Definitely not ❒ Probably not ❒ Probably ❒ Definitely

72. What score would you give the A&E?

❒ 0 ❒ 1 ❒ 2 ❒ 3 ❒ 4 ❒ 5 ❒ 6 ❒ 7 ❒ 8 ❒ 9 ❒ 10

73. Did you get the care you expected from the A&E?

❒ No, not at all ❒ A bit ❒ A great deal ❒Yes, completely

74. How would you describe your overall health?

❒ Outstanding ❒ Very well ❒ Well ❒ Moderate ❒ Poor

75. How old are you?

❒ 0 to 11 years ❒ 12 to 17 ❒ 18 to 24 ❒ 25 to 34 ❒ 35 to 44 ❒ 45 to 54 ❒ 55 to 64 ❒ 65 to 74 ❒ 75 or older

76. Are you a man or a woman?

❒ Man ❒ Woman

77. What is your highest level of education?

❒ No education ❒ Low education ❒ Middle education ❒ High education

78. Where were you born?

❒ Netherlands ❒ Indonesia / Former Dutch Indies ❒ Suriname ❒ Morocco ❒ Turkey

❒ Germany ❒ Netherlands Antilles ❒ Aruba ❒ Other, namely ………

79. Where was your father born?

❒ Netherlands ❒ Indonesia / Former Dutch Indies ❒ Suriname ❒ Morocco ❒ Turkey

❒ Germany ❒ Netherlands Antilles ❒ Aruba ❒ Other, namely ………

80. Where was your mother born?

❒ Netherlands ❒ Indonesia / Former Dutch Indies ❒ Suriname ❒ Morocco ❒ Turkey

❒ Germany ❒ Netherlands Antilles ❒ Aruba ❒ Other, namely ………

81. What language do you mostly speak at home?

❒ Dutch ❒ English ❒ Friesian ❒ Dutch dialect ❒ Indonesian ❒ Sranan (Surinaams)

❒ Moroccan-Arabian ❒ Turkish ❒ German ❒ Papiaments (Dutch Antilles) ❒ Other, namely

82 Did someone help you fill in this questionnaire? (parents or guardians of children under 12 can tick yes here)

❒ No 🡪 Go to *question 84* ❒ Yes

83. How did this person help you (several answers possible)

❒ Read the questions out ❒ Wrote my answers down ❒ Answered the questions on my behalf ❒ Translated the questions into my language ❒ I am a parent or guardian of a child under 12 years ❒ Helped in another manner, namely……….

84. What would you change in the care you received in the A&E at (*Insert name instiution)*?

**Importance study**

The importance questions have the response categories:

A. Not important; B. Of some importance; C. Important; D. Extremely important

1. How important is the availability if the record (data) of your previous visit to the A&E to you?
2. How important is the signposting to the A&E of the hospital to you?
3. How important is the travelling time to the A&E of the hospital to you?
4. How important is finding a parking space near to the A&E to you?
5. How important is finding the A&E in the hospital to you?
6. How important is having enough privacy at the reception counter when you explain your health problem to you?
7. How important is the politeness of the reception staff member to you?
8. How important is it to you that the reception staff member treats you seriously?
9. How important is it to you that the reception staff gives you information on what to expect

during your visit to the A&E?

1. How important is the waiting time until you first speak to a healthcare professional to you?
2. How important is it to you that the nurse tells you how quickly you needed to be helped with your health problem?
3. How important is to you that the nurse tells you the order you and the other patients in the waiting room would be helped?
4. How important is the second waiting time before treatment to you?
5. How important is it to you that the total waiting time before treatment is not a problem?
6. How important is it to you that more serious patients were treated first?
7. How important is it to you that healthcare professionals ask you if you are in pain?
8. How important is it to you that you receive medication to reduce the pain?
9. How important is the help of healthcare professionals to control your pain?
10. How important is the privacy during your examination in the treatment room to you?
11. How important is it to you that if you need help, you receive it as quickly as you want?
12. How important is it to you that you receive the help you need?
13. How important is it to you that the care provider in the treatment room gives you information on the steps in your treatment?
14. How important is it to you that the care provider explains the results of these tests in an understandable manner?
15. How important is it to you that you can decide about your treatment?
16. How important is it to you that you are asked to consent to your treatment?
17. How important is the politeness of the healthcare professionals to you?
18. How important is it to you that the healthcare professionals listen to you attentively?
19. How important is it to you that the healthcare professionals have enough time for you?
20. How important is it to you that the healthcare professionals take you seriously?
21. How important is it to you that the healthcare professionals talk in front of you as if you aren’t there?
22. How important is it to you that the healthcare professionals explain your health problem in an understandable manner?
23. How important is it to you that you don’t receive contradictory information of the healthcare professionals?
24. How important is the cooperation of the healthcare professionals with each other to you?
25. How important is trust in the expertise of the healthcare professionals in the A&E to you?
26. How important is it to you that you have to tell the same story several times?
27. How important is it to you that the healthcare professionals explain the aim of the new medicines in an understandable manner?
28. How important is it to you that the healthcare professionals inform you of side-effects to which you had to pay attention?
29. How important is it to you that the healthcare professionals tell you when you can resume your usual activities, such as eating or walking?
30. How important is it to you that the healthcare professionals tell you which danger signals you should watch out for after leaving the A&E?
31. How important is it to you that your healthcare professionals tell you who to contact if you are worried about your health problem after leaving the A&E?
32. How important is it to you that the healthcare professionals tell you that your general practitioner will be informed about your visit to the A&E?
33. How important is it to you that you receive a referral letter for your general practitioner?
34. How important is it that the healthcare professionals explain how to make a follow-up appointment?
35. How important is it to you that the atmosphere (planning) of the waiting room is pleasant? (magazines/television/chairs)?
36. How important is it that if you so wish, you can obtain something to eat or drink in the A&E?
37. How important is the hygiene in the A&E to you?
38. How important is a quiet environment in the A&E to you?
39. How important is feeling safe during your stay in the A&E to you?
40. How important is it to you that if you are accompanied by others (partner/family/friends), they get information on you?
41. How important is it to you that you receive the care you expect from the A&E?
